# Removal of Acetaminophen Drug from Wastewater by Fe_3_O_4_ and ZSM-5 Materials

**DOI:** 10.3390/nano13111745

**Published:** 2023-05-26

**Authors:** Florinela Pirvu, Cristina Ileana Covaliu-Mierlă, Gina Alina Catrina

**Affiliations:** 1 Faculty of Biotechnical Systems Engineering, Politehnica University of Bucharest, 313 Splaiul Independentei, 060042 Bucharest, Romania; florinela_pirvu@yahoo.com; 2 National Research and Development Institute for Industrial Ecology ECOIND, 71-73, Drumul Podu Dambovitei Street, 060652 Bucharest, Romania

**Keywords:** acetaminophen, Fe_3_O_4_, ZSM-5, adsorption, capacity isotherms

## Abstract

Adsorption of toxic compounds from water using zeolites and magnetite was developed due to the various advantages of their applicability. In the last twenty years, the use of zeolite-based compositions in the form of zeolite/inorganic or zeolite/polymer and magnetite has been accelerated for the adsorption of emergent compounds from water sources. The main adsorption mechanisms using zeolite and magnetite nanomaterials are high surface adsorption, ion exchange capacity and electrostatic interaction. This paper shows the capacity of Fe_3_O_4_ and ZSM-5 nanomaterials of adsorbing the emerging pollutant acetaminophen (paracetamol) during the treatment of wastewater. The efficiencies of the Fe_3_O_4_ and ZSM-5 in the wastewater process were systematically investigated using adsorption kinetics. During the study, the concentration of acetaminophen in the wastewater was varied from 50 to 280 mg/L, and the maximum Fe_3_O_4_ adsorption capacity increased from 25.3 to 68.9 mg/g. The adsorption capacity of each studied material was performed for three pH values (4, 6, 8) of the wastewater. Langmuir and Freundlich isotherm models were used to characterize acetaminophen adsorption on Fe_3_O_4_ and ZSM-5 materials. The highest efficiencies in the treatment of wastewater were obtained at a pH value of 6. Fe_3_O_4_ nanomaterial presented a higher removal efficiency (84.6%) compared to ZSM-5 nanomaterial (75.4%). The results of the experiments show that both materials have a potential to be used as an effective adsorbents for the removal of acetaminophen from wastewater.

## 1. Introduction

A very important element of the ecosystem, water is an essential component of life. Due to the increase of the population and the standard of living, there has been an increasing demand for water. The quality of water resources deteriorates daily due to the use in significant quantities of chemical compounds in hospitals, drug factories as well as in the chemical industry. Water pollution [1] with drugs and pharmaceutical residues is growing. These so-called emerging pollutants can, due to their nature, lead to endocrine, hormonal and genetic disorders to humans. In different sources of water were found pharmaceuticals from almost all classes of drugs, namely: anti-inflammatory, antibiotic, antidiabetic, antidepressant, antiepileptic, antihistamine, antipsychotic, β-blockers, cytostatic, gastrointestinal, regulators of lipids, steroids [2]. 

In recent years, the properties of nanomaterials have been studied for their use in the removal of pharmaceutical products from wastewater, which can become an interesting field of research [3,4,5]. Among these properties, a high adsorption capacity can be highlighted, especially due to their large surface area and the large number of active sites for interaction with different pollutants found in water. Furthermore, adsorbents with specific functional groups have been developed to improve the adsorption capacity of these materials [6]. Adsorption techniques can allow the treatment of water containing acetaminophen or its degradation products and could have the advantages of simplicity and cost effective [7].

The studies that present the use of nanomaterials also include some advantages compared to other methods used for water treatment. Current wastewater treatment technologies cannot completely remove pharmaceutical residues and their metabolites, therefore, for the treatment of wastewater, a number of factors such as the quality standards to be achieved, efficiency and cost should be taken into account. Also, the conditions that could be respected in choosing a wastewater treatment technology can be listed as follows: low cost, flexibility of treatment, reuse of agents used in treatment, environmental safety as well as final efficiency [5,6,7].

Due to the low cost and chemical rezistant to toxic contaminants, the adsorption process can be easily used and numerous materials have been studied for the removal of these pollutants (e.g., activated carbon, graphene oxide, carbon nanotubes). The adsorption behavior can be influenced by the type of the pollutant. The adsorbant surface, the size of the particles, the interaction between the adsorbate and the absorbant, are factors that can influence the adsorption process [8,9].

Pharmaceutical compounds are used in the manufacture of drugs used to treat various ailments, fight almost all infections, and reduce uncomfortable symptoms. Not all medicines are completely metabolized in the body and for this reason they can be excreted in the sewage system through urine and fasces. Excretion through the lungs occurs only with highly volatile gaseous compounds [9,10].

**Figure 1:**
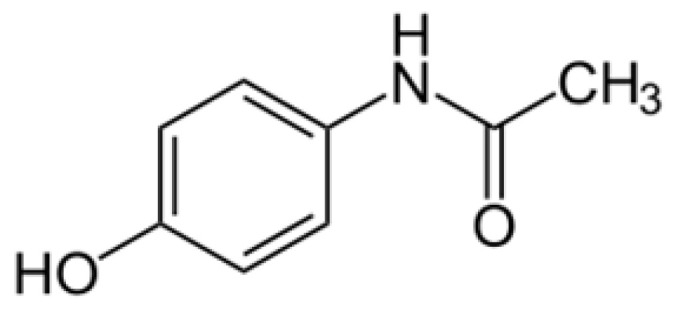
Structural formula for Acethaminophen.

Varous researches have shown that numerous pharmaceutical compounds pass through sewage treatment plants (STP) and can end up in the aquatic environment. Acetaminophen (Figure 1) is widely administered, as it is indicated in the symptomatic treatment of pain of mild to moderate intensity, occurring after various types of procedures, as well as sore throat, post-vaccination pain and menstrual pain [11,12].

Generally, research for the detection and determination of pharmaceutical compounds in the environment is growing but methodologies must be established for the development of monitoring systems and constant data collection, because it is important to know the influence of these pharmaceutical residues, being known that there are special programs for environmental risk assessment. 

Degradation products of paracetamol from different types of wastewaters, such as urban wastewater and those from pharmaceutical production, can reach surface waters. Being one of the most common emerging pollutants, due to its frequent use, paracetamol [13] can be considered toxic to aquatic organisms (endocrine disrupting effect on some fish) even when it is in low concentrations in the environment. In some species of crustaceans, the concentrations of paracetamol existing in the studied waters could lead to an increase in mortality due to developmental abnormalities as well as changes in sexual hormones [14].

The potential of ecological chemistry may be another area for further research that would reduce or even eliminate the dangers of pharmaceutical compounds reaching the environment [15,16,17].

In the literature, Jayasree and Remya [18] removed paracetamol by photocatalysis from wastewater using TiO_2_ on aluminosilicate which was recovered from waste LED panels. After 30 min of UV irradiation, a removal efficiency of 99% was measured at an initial concentration of the compound of interest of 2.74 mg/L and using an amount of TiO_2_ of 2.71 g/L at a pH of 9.5.

Tepe [19] studied the removal of paracetamol from water by an oxidative method. It used manganese oxide (OMS-2), persulfate (PS) and achieved a 99.5% removal of the studied pollutant.

The degradation method of paracetamol used by Periyasamy and Muthuchamy [20] was electrooxidation. Graphite was used as the anode, 20 mg/L was the initial concentration of paracetamol, the removal efficiency was over 90% after 240 min, the current density used was 5.1 mA/m^2^, and the electrolyte used was Na_2_SO_4_ with a concentration of 0.1 M at pH 4.

The pH of the solution, the mass of the adsorbent, the contact time, the temperature and the surface area of adsorbent can influence the interaction between the adsorbent and the pollutants from water. The ionization of functional groups on the specific surface and the selection of metal ions are affected by the pH value. It is considered that pH plays an essential role in adsorption in aqueous solutions, because it affects the character of each removed ionic species and adsorbents (when the pH of the solution exceeds 7, the adsorption phenomena disappear and change into the precipitate) [20,21]. Krishnan et al. [22] found that the pH of the wastewater decreases when the acid group on the surface of the adsorbent increases. If the pH of a water is higher than the pH of the adsorbent, it provides negative surface charge information by adsorbing cationic species, which shows that the pH of a solution has a significant effect on the adsorbent, implicitly on the adsorption capacity [21].

The appropriate contact time can be described as the time required to reach equilibrium. The adsorption efficiency depends on the particle size and the specific surface area of the adsorbent. The surface area increases as the particle size decreases. An adsorbent must be mechanically stable, must have a high specific surface area, and be economically feasible [22,23,24]. Matsui et al. [25] studied the particle size of different zeolites and reported that particle uptake was 10% higher for 75–100 µm sizes than 150–250 µm.

The absorption capacity [24,25,26] of a solid adsorbent for a certain concentration of adsorbent in a solution is influenced by the mass of the adsorbent. Elsayed and Osman [21] and Mahmudi and Arsad [27] show that the availability of exchange sites on the specific surface of the adsorbent can influence the effect of the adsorbent dose on the adsorption capacity. Before the saturation point, the maximum removal of adsorbed ions from the solution was achieved. The use of several types of adsorbents may not affect the removal process. For the removal of pharmaceutical products, the possible mechanisms can be hydrophobic interactions, electrostatic interactions and hydrogen bonds [27].

High adsorption capacity of adsorbents with Fe_2_O_3_ content is due to the following factors: the present Fe_2_O_3_ improved the surface and provided a bridge for ion transport, the present Fe_2_O_3_ in the carbon network may have anchored the adsorption capacity of the material by providing a stable nanostructure, uniformly distributed Fe_2_O_3_-containing magnetic materials acted as conductive bridges to potentially improve the adsorbed process by allowing surface active sites to participate in adsorption/desorption activities efficiently, the super hydrophilic nature of magnetite-containing adsorbent materials allowed the aqueous solution to penetrate deeper inside the sites, shortening the duration of the adsorption process [27,28,29].

This study presents the comparison of magnetite and zeolite nanomaterials for the removal of acetaminophen from wastewater.

The effects of various experimental parameters on acetaminophen adsorption were investigated by batch adsorption study: acetaminophen concentration, pH, adsorbent dose, contact time. The procedure for the determination of acetaminophen was internally validated.

## 2. Materials and Methods

### 2.1. Chemicals and Reagents

The purity of the analytical standard (acetaminophen) purchased from Sigma Aldrich, St. Louis, MO, USA, was in the range of 98–100%. The 37% HCl was also purchased from Sigma Aldrich St. Louis, MO, USA.

The nanomaterials used for this study were magnetite (Fe_3_O_4_) and zeolites (ZSM-5), and the methods of obtaining them are presented elsewhere [16,17].

### 2.2. Batch Adsorption Studies

**Figure 2:**
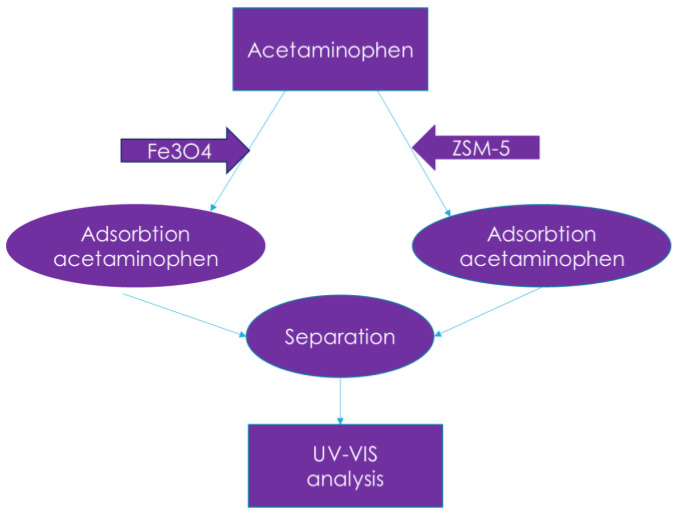
Schematic presentation of the experimental process.

A UV-Vis spectrophotometer (Specord 200 Plus, Analytic Jena, Jena, Germany) was used to measure the concentration of acetaminophen in the solution (Figure 2).

The acetaminophen synthetic solutions of varying initial concentrations (50–280 mg/L) were prepared and analyzed at a wavelength of 302 nm. The absorbance values were plotted against corresponding acetaminophen concentrations to obtain the calibration curve, and the unknown concentrations of acetaminophen solutions were determined from this curve. 

**Figure 3:**
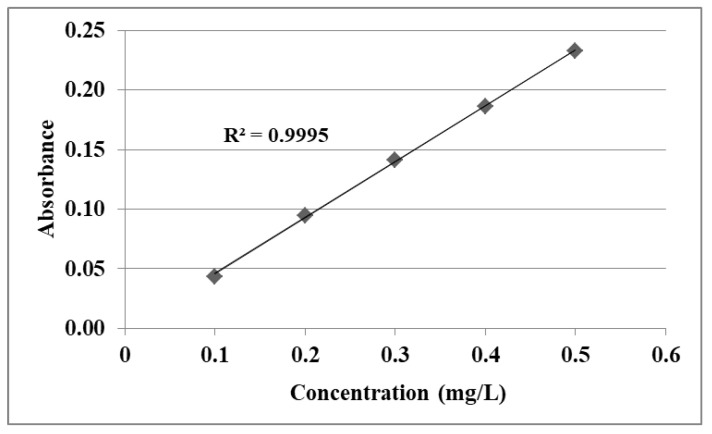
Acetaminophen calibration curve.

For the quantification of acetaminophen, a calibration plot was made in the range of 0.10–0.50 mg/L (Figure 3). The linearity studies are presented in Table 1.

### 2.3. Internal Validation Method

The performance parameters for the validation method were limit of detection and limit of quantitation, which implies 5 independent fortified blank solutions (0.05 mg/L); repeatability, which implies 10 independent standard solutions of 0.25 mg/L concentration; intermediate precision, which implies 10 independent standard solutions of 0.35 mg/L and expanded uncertainty, which implies bias and precision. The method developed in this study was validated in accordance with the ICH guidelines [30]. There were 10 determinations for repeatability and precision, quantification limit, detection limit and extended uncertainty for acetaminophen in the range of 0.10–0.50 mg/L using two dissolved media [30].

Table 2 shows the performance parameters for acetaminophen using an internal method obtained with 0.1 M HCl as the dissolution medium.

### 2.4. The Batch Adsorption Study

The adsorption studies have implied a few parameters, such as pH, contact time, dosage of adsorbent and initial acetaminophen concentration. The batch adsorption study was carried out by adding 0.2 g and 0.5 g of adsorbent to a series of 250-mL conical flasks containing a 100 mL wastewater containing acetaminophen. The flasks were homogenized in a mechanical shaker, and the samples were collected at predetermined time intervals. The samples were centrifuged for 5 min and analyzed by spectrophotometry to determine the acetaminophen concentration in the wastewater solution. The amount of acetaminophen adsorbed was calculated from the relationship as follows:

(1) $$q_{e}\left(\mathrm{mg}/g\right)=\frac{\left(C_{0}-\mathrm{Ce}\right)}{C_{0}}100$$

where ***q_e_*** is adsorption capacity at equilibrium (mg/g); *C*_0_ is the initial acetaminophen concentration (mg/L); *Ce* is the equilibrium acetaminophen concentration (mg/L); *V* is the volume of wastewater solution (L) and m is the amount of adsorbent (g).

The removal efficiency was calculated with the following formula:

(2) $$R(\%)=\frac{\left(C_{0}-\mathrm{Ce}\right)}{C_{0}}100$$

where ***R*** is removal efficiency (%); *C*_0_ is the initial acetaminophen concentration (mg/L); *C_e_* is the equilibrium acetaminophen concentration (mg/L).

### 2.5. Adsorption Isotherms

The acetaminophen adsorption process onto Fe_3_O_4_ and ZSM-5 was described by using the Langmuir and Freundlich equation. Optimizing operation conditions in designing water/wastewater treatment is an important issue that implies the adsorption process.

Therefore, establishing the adsorption capacity of adsorbent materials with different isotherm models allows to design a treatment process and to optimize operating conditions adequately. Table 3 describes the Langmuir and Freundlich equations.

## 3. Results and Discussions

The task was to determine the acetaminophen concentration in solution (applying spectrophotometry method) before and after adsorption.

### 3.1. The Effect of Solution pH on the Quantity of Acetaminophen Removed by Fe_3_O_4_ and ZSM-5

**Figure 4:**
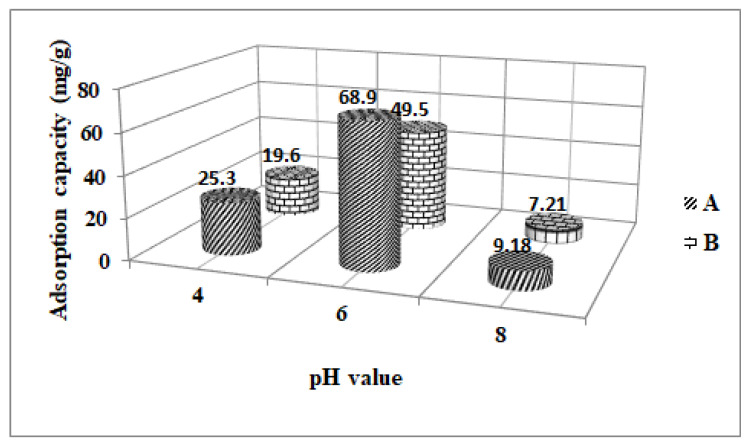
The effect of pH solution on the adsorption capacity of acetaminophen removed using **A** (Fe_3_O_4_) and **B** (ZSM-5).

The effect of pH on Fe_3_O_4_ and ZSM-5 adsorption capacity was obtained by using acetaminophen wastewater solutions adjusted to pH 4–8 (Figure 4). The adsorption process is influenced by a series of factors such as pH value, time and initial acetaminophen concentration. The effect of pH is due to the fact that at different pH values, the adsorbent surface has a negative or positive charge and the pH value affects the acetaminophen speciation in the aqueous solution. Consequently, repulsive or attraction forces between the pollutant and the adsorbent surface can occur. Moreover, the majority of the industrial effluents and wastewater with acetaminophen loading have different pH values depending on the type of industrial activities performed. Thus, it is necessary to carry out studies to establish the influence of the pH value on acetaminophen adsorption by Fe_3_O_4_ and ZSM-5 nanomaterials. 

### 3.2. The Effect of Dosage Adsorbent on the Removal Efficiency of Fe_3_O_4_ and ZSM-5 

**Figure 5:**
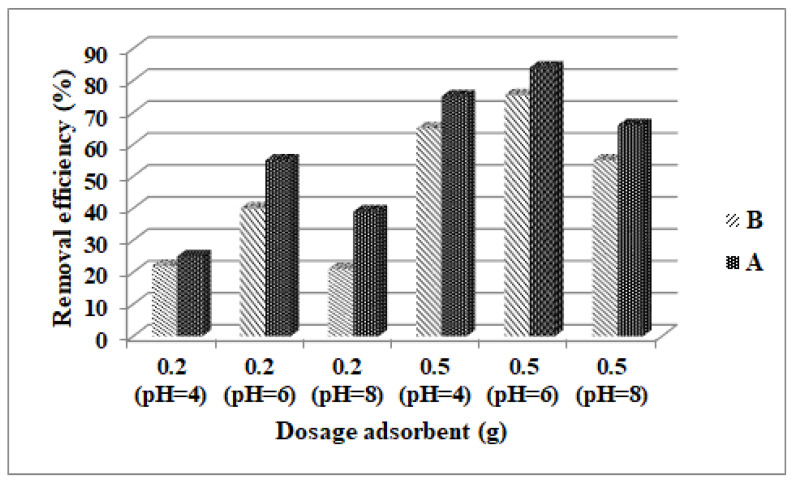
The effect of dosage adsorbent on the removal efficiency of acetaminophen using **A** (Fe_3_O_4_) and **B** (ZSM-5).

In this study, two quantities of adsorbent material were used, 200 mg and 500 mg for both materials (Fe_3_O_4_ and ZSM-5). Adsorption studies were performed using two initial concentrations of acetaminophen: 50 and 280 mg/L acetaminophen for both tested materials. The used procedures are presented in Table 1, Table 2, Table 3 and Table 4. Figure 5 represents the graph results obtained using both adsorbent materials.

The higher removal efficiency was obtained at pH 6, using 0.5 g as adsorbent material. Fe_3_O_4_ obtained the highest results for removal efficiency (84% at pH = 6) in comparison to ZSM-5, which presented a removal efficiency of 75% (Figure 5).

### 3.3. The Effect of Contact Time on the Removal Efficiency of Fe_3_O_4_ and ZSM-5

The results presented in Figure 5 revealed that for both materials tested, the optimum contact time was 8 h and adsorption was faster in the first 4 h. 

**Figure 6:**
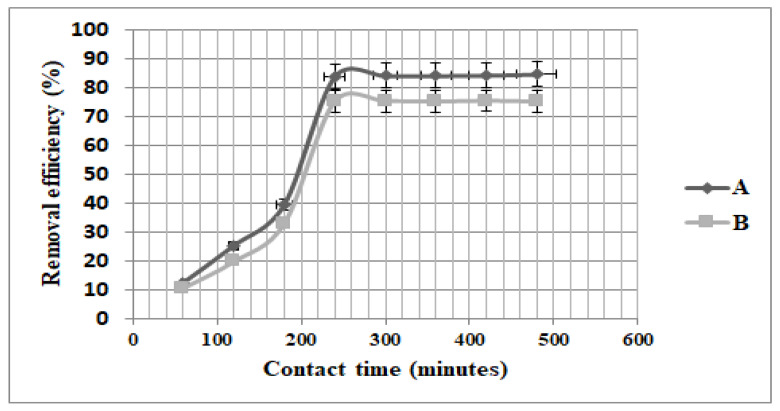
The effect of contact time on acetaminophen removal efficiency from wastewater using **A** (Fe_3_O_4)_ and **B** (ZSM-5) nanomaterials.

After that, a slow increase in adsorption was observed until adsorption equilibrium was reached. In the first fast adsorption stage, all the adsorption sites of nanomaterial are available and they were occupied by the acetaminophen. As the acetaminophen adsorption continues more adsorption sites are occupied successively, and consequently the number of free sorption sites decreases and the adsorption becomes slower. Different behavior in the adsorption process in the function of the two nanomaterials used for their preparation has been observed. Fe_3_O_4_ nanomaterial presents higher removal efficiency (84.6%) compared to ZSM-5 nanomaterial (75.4%) as can be seen in Figure 6, with error bars of 5%. All the values recorded for Fe_3_O_4_ and ZSM-5 fall within the standard deviation of 5.

### 3.4. Langmuir and Freundlich Adsorption Isotherms

Figure 6 presents the linear forms of equilibrium isotherms. They have been used to determine values of adsorption parameters.

**Figure 7:**
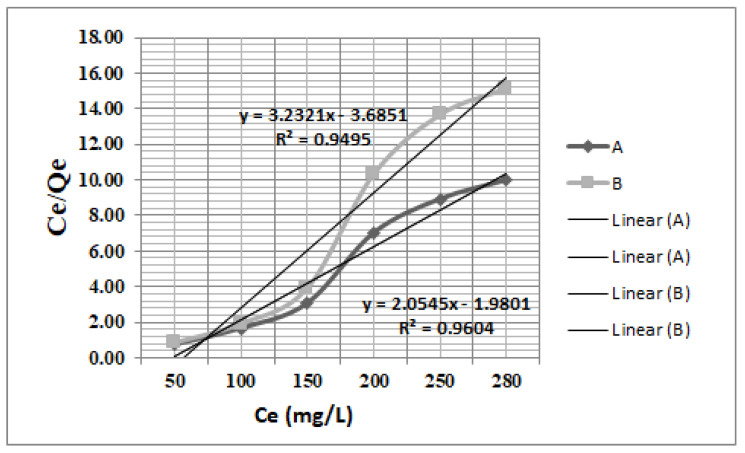
Langmuir linearized isotherm for acetaminophen adsorption using **A** (Fe_3_O_4)_ and **B** (ZSM−5) nanomaterials.

**Figure 8:**
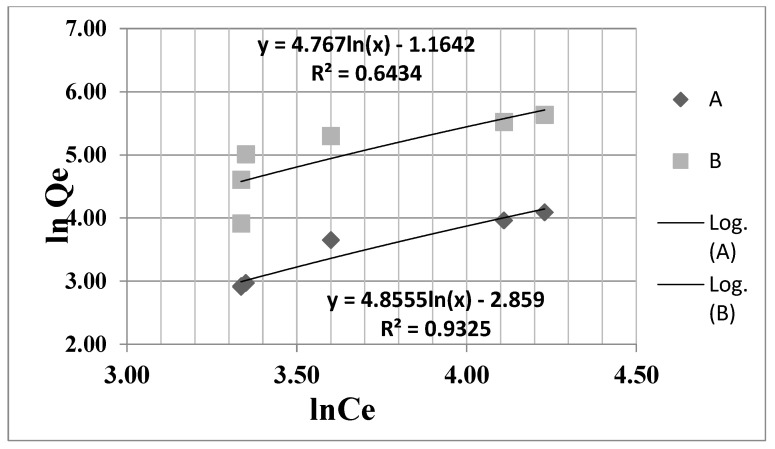
Freundlich linearized isotherm for acetaminophen adsorption using **A** (ln Qe Fe_3_O_4_) and **B** (ln Qe ZSM−5).

Experimental data presented in Figure 7 and Figure 8, and Table 4 revealed that the Langmuir isotherm model fits very well with the experimental data for all adsorbent materials tested.

Table 4 and Figure 7 and Figure 8 revealed that the Langmuir equation (correlation coefficient R^2^ = 0.964 and R^2^ = 0.953, respectively) describes the adsorption isothermal behaviors for both Fe_3_O_4_ and ZSM-5 nanomaterials tested well. The Langmuir constant R_L_ is in the range (0–1), indicating that the retention process of acetaminophen is favorable using these nanomaterials. The Freundlich isotherm presents a correlation coefficient (R^2^ = 0.9325 for Fe_3_O_4_ and R^2^ = 0.6434 for ZSM-5) much lower than the Langmuir isotherm, as can be seen in Figure 7 and Figure 8. 

It can be seen from Figure 4 that the adsorption of acetaminophen decreases with the increase in the pH of the wastewater solution, and the maximum acetaminophen adsorption is obtained at a pH of 6. At a lower solution pH, the acetaminophen molecules do not get protonated into other molecules, which leads to a higher adsorption capacity. On the other hand, at higher pH values, there is competitive adsorption between acetaminophen and (OH^−^) molecules leading to lower adsorption capacity. On the contrary, at acidic pH, some of the negatively charged ions are neutralized, and the acetaminophen molecules also remain dissociated, leading to a higher adsorption capacity. Therefore, the acetaminophen adsorption efficacy of wastewater can be enhanced in an acidic medium. In the present investigation, the maximum adsorption of acetaminophen was obtained for a pH of 6 of the wastewaters. 

## 4. Conclusions

Two adsorbent materials (Fe_3_O_4_ and ZSM-5) were used for the removal of acetaminophen from wastewater. The highest adsorption capacity (68.9 mg/g) and the highest removal efficiency (84.6%) was obtained for the Fe_3_O_4_ at 6 value of pH. 

The other material ZSM-5 obtained an adsorption capacity of 49.5 mg/g and a removal efficiency of 75% for the wastewater polluted with the acetaminophen. The difference between the two adsorbent materials studied is based on active surface, porosity and the method which were obtained the adsorbent materials. 

The adsorption data were evaluated using two mathematical models (Langmuir and Freundlich). It was found that the adsorption characteristics were well predicted by Langmuir adsorption isotherm. The equilibrium parameter (R_L_) for Langmuir isotherm was in the range of 0–1 which indicates that the adsorption process is favorable for Fe_3_O_4_ and ZSM-5 nanomaterials. 

Adsorption studies to remove emerging pollutants from the wastewater are limited and strategies could be developed to improve the efficiency of treatment plants.

The proposed method is simple, sensitive, rapid, specific, could be applied and could be improved for monitoring of acetaminophen and its by-products from wastewater. 

## Figures and Tables

**Table 1:** Results obtained for linearity studies.

Compound	Linearity Range (mg/L)	Regression Equations	R^2^	λ (nm)
Acetaminophen dissolved in 0.1 M HCl	0.10–0.50	y = 0.4591x + 0.0004	0.9994	302

**Table 2:** Performance parameters of acetaminophen.

No.	Acetaminophen		
	Performance Parameters	U.M.	Obtained Results
1	Precision	mg/L	0.03
2	Detection limit (LOD)	mg/L	0.04
3	Quantification limit (LOQ)	mg/L	0.12
4	Extended uncertainty	%	17.5

U.M.—measurement units.

**Table 3:** Langmuir and Freundlich equation.

Langmuir Equation	Observations
(3) $\frac{C_{e}}{q_{e}}=\frac{1}{q_{max}}C_{e}+\frac{1}{q_{max}}\frac{1}{K_{L}}$	***Ce***—is the equilibrium concentration of acetaminophen, mg/L; ***q_e_***—is the amount of acetaminophen adsorbed per gram of the asorbent at equilibrium mg/g; ***q*_max_**—is the maximum quantity, mg/g; ***K_L_***—is the Langmuir isotherm constant, L/mg; ***q*_max_** and ***K_L_*** were obtained from the slope and intercept of the plots.
(4) $R_{L}=\frac{1}{1+K_{L}}\frac{1}{C_{0}}$	***R_L_***—of a dimensionless constant separation factor or equilibrium parameter ***R_L_***—value indicates that the process is unfavorable if *R_L_* > 1; linear if *R_L_* = 1; favorable if 0 < *R_L_* < 1 and irreversible if *R_L_* = 0; ***K_L_*** is the Langmuir constant; ***C*_0_**—is the initial concentration, measured in mg/L.
**Freundlich Equation**	**Observations**
(5) $\ln q_{e}=lnK_{F}+\frac{1}{n}lnC_{e}$	***K_F_*** is Freundlich constant which represents sorption capacity and ***n*** is the Freundlich constant that shows sorption intensity. ***K_F_*** and ***n*** were calculated from the intercepts and slopes of the Freundlich plots. ***Ce***—is the equilibrium concentration of acetaminophen, mg/L;

**Table 4:** Langmuir and Freundlich adsorption parameters.

Adsorbent Material	Langmuir Parameters				Freundlich Parameters		
	Q_max_ (mg/g)	K_L_ (L/mg)	R_L_	R^2^	K_F_ (L/g)	1/n	R^2^
Fe_3_O_4_	68.9	0.149	0.79	0.9644	42.13	0.356	0.9325
ZSM-5	49.5	0.103	0.86	0.9530	33.89	0.314	0.6434

## Data Availability

Not applicable.
